# Ultrasound- and circumference-based quadriceps mass is an independent predictor of 28-day mortality in critically ill patients

**DOI:** 10.3389/fnut.2026.1751365

**Published:** 2026-04-07

**Authors:** Ying Liu, Shengming Ye, Hua Xiao, Dan Su

**Affiliations:** 1 Department of Neurology, Beijing Shunyi Hospital, Beijing, China; 2 Department of Emergency, Beijing Chaoyang Hospital, Capital Medical University, Beijing, China; 3 Department of Scientific Research Office, Beijing Shunyi Hospital, Beijing, China; 4 Department of Geriatrics, Liangxiang Hospital of Beijing Fangshan District, Beijing, China

**Keywords:** critically ill patients, mortality, quadriceps circumference, quadriceps muscle thickness, ultrasonography

## Abstract

**Background:**

Skeletal muscle mass is a key indicator of physiological reserve in critical illness.

**Objective:**

This study aimed to evaluate whether quadriceps mass assessed by bedside methods predicts 28-day mortality in critically ill patients.

**Methods:**

In this prospective study of 603 critically ill adults, we measured quadriceps thickness by ultrasonography under minimal transducer pressure (QT-min) and maximal transducer pressure (QT-max), quadriceps circumference (QC), and mid-upper arm circumference (MUAC) at admission. Cox regression was used to analyze the association between quadriceps thickness and 28-day mortality. Interaction and subgroup analyses were conducted for age, sex, BMI, mechanical ventilation, number of organ supports and vasopressor use.

**Results:**

The 28-day mortality rate was 21.06% (127/603). After adjustment in Model 3, QC (HR 0.95 per 1-cm increase, 95% CI 0.91–1.00), QT-min (HR 0.63 per 1-cm increase, 95% CI 0.42–0.92), and QT-max (HR 0.42 per 1-cm increase, 95% CI 0.20–0.85) remained independent protective factors for mortality, while MUAC do not. Significant interactions were found for QT-min with vasopressor use and organ support (*q* < 0.05), with protective effects observed only in patients without these conditions.

**Conclusion:**

Bedside quadriceps mass assessment independently predicts 28-day mortality in critically ill patients, supporting its use for early risk stratification. In patients requiring vasopressors or organ support, the prognostic value of QT-min was attenuated, suggesting it may be susceptible to illness severity. However, given the small size of these subgroups, this finding warrants a cautious interpretation.

## 1 Introduction

Accurate assessment of body composition and nutritional status in critically ill patients enables better risk stratification, facilitates early identification of patients at high mortality risk, and ultimately informs treatment optimization and clinical decision-making. Although established disease severity scoring systems such as APACHE II and SOFA are widely used in clinical practice and incorporate a range of physiological and laboratory parameters, they often fail to reflect the significant role of nutritional and functional reserves in determining survival outcomes.

Skeletal muscle mass serves as a key indicator of physiological reserve (1). During critical illness, accelerated protein catabolism leads to rapid muscle wasting, with losses of approximately 2% per day during the first week of ICU admission (2, 3). While current guidelines recommend protein intake of ≥1.2 g/kg/day for critically ill patients (4), early higher protein intake has not been shown to improve outcomes in those receiving invasive ventilation (5, 6). Notably, previous studies have identified an independent association between early enteral nutrition and reduced in-hospital mortality in patients with sarcopenia—an effect not observed in individuals with a modified NUTRIC score >4 or abnormal BMI (7). These findings emphasize that skeletal muscle loss is a critical factor influencing outcomes and guiding nutritional and rehabilitation strategies in critically ill patients.

A growing body of evidence supports low skeletal muscle mass as an independent predictor of mortality in critical illness. A study of 240 critically ill patients evaluated by abdominal computerized tomography (CT) imaging demonstrated that lower skeletal muscle volume was independently associated with increased mortality, regardless of APACHE II score or gender (8). A recent systematic review and meta-analysis including 38 studies with 6,891 patients demonstrated that critically ill patients with low skeletal muscle mass have a significantly higher mortality risk (9). However, it is noteworthy that 89.4% of the included studies employed CT for muscle assessment.

Although CT-based measurements provide accurate assessment of muscle area and density, their routine use in critically ill patients is limited by practical constraints such as cost, time, transport-related risks, and radiation exposure. These limitations have driven interest in bedside alternatives. Bedside muscle assessment offers a practical and safe approach, eliminating transport-related risks. Ultrasound, as a non-invasive and portable tool, has been shown to accurately and reliably capture dynamic muscle changes in this population (10–13). A 2023 systematic review found that ultrasound was the most commonly used method for muscle mass, employed in 85% of studies (3). Despite meta-analyses demonstrating an association between low muscle mass and mortality, most existing studies rely on complex imaging techniques such as CT or MRI, with a notable lack of systematic evaluation of bedside alternatives like ultrasound (9).

Furthermore, the comparative prognostic value of different bedside measurements, such as manual circumference and ultrasound-derived thickness parameters, has not been rigorously evaluated. Standardization of ultrasound measurement protocols also warrants investigation (14), as variations in the pressure applied by different operators may influence the accuracy and reproducibility of muscle measurements. Muscle compressibility can be altered by pathological conditions such as edema (15), fibrosis, or fatty infiltration (16), and muscle thickness measured under different compression protocols may therefore provide distinct prognostic information. However, the clinical significance of this distinction remains unexplored.

Therefore, this study aimed to investigate the association between muscle mass and 28-day mortality in critically ill patients using multiple bedside methods, including manual measurements (mid-upper arm circumference and quadriceps circumference) and ultrasonography with two distinct pressure protocols. We further assessed whether low muscle mass detected by these bedside-adaptable approaches independently predicted increased mortality risk across different patient subgroups.

## 2 Methods

### 2.1 Study design and participants

This is a longitudinal observational population-based study. Critically ill patients were consecutively recruited from the Emergency Rescue Room of Beijing Chaoyang Hospital, Capital Medical University, between March 2023 and December 2024. All patients aged ≥18 years admitted to the emergency intensive care unit during the study period were screened for eligibility. The exclusion criteria were as follows: (1) inability to cooperate with the study procedures (e.g., any condition precluding muscle measurement or follow-up), (2) pregnancy or lactation, (3) pre-existing psychiatric disorders, and (4) incomplete clinical data. Written informed consent was obtained from all participants or their legal representatives. The study protocol was approved by the Institutional Ethics Committee of Beijing Chaoyang Hospital (Approval No. 2022-Ke-430). The study was not pre-registered on any platform, nor was the sample size pre- determined through prior registration. However, to assess sample size adequacy, we performed a *post hoc* power analysis for all statistical tests using the pwr package in R. With a two-sided *α* of 0.05, our sample size provided >99.99% power, supporting the reliability of the findings. The study protocol was developed and is available from the corresponding author upon reasonable request.

### 2.2 Follow-up and outcome ascertainment

Patients were followed up from the day of hospital admission until death or day 28, whichever occurred first. Vital status at 28 days was determined through electronic medical records. For patients discharged alive before day 28, telephone follow-up on day 28 was conducted to ascertain vital status. For those who had died, the exact date of death was documented. Patients who were lost to follow-up or discontinued participation for any reason were not included in the final analytical cohort. All patients included in the final cohort were successfully followed until death or day 28.

### 2.3 Exposure measures

The primary exposure variables comprised quadriceps thickness (assessed by ultrasonography), quadriceps circumference (QC), and mid-upper arm circumference (MUAC). Muscle assessment was performed within 2 days after admission. Limb circumference measurements were obtained using a non-elastic tape measure. For MUAC, the midpoint between the left acromion and olecranon was identified and measured. Similarly, the QC was measured at the midpoint between the left anterior superior iliac spine and superior border of the patella. Each measurement was performed twice, and the average was recorded. If the two measurements differed significantly (>2 cm), a third measurement was taken to ensure accuracy.

Muscle ultrasound assessments were performed by a licensed physical therapist with over 5 years of experience in sonographic measurement and image analysis. Quadriceps measurements were performed on the left leg with the patient in the supine position, knees fully extended and relaxed, and toes pointing upward. The thicknesses of the rectus femoris and vastus intermedius muscles were evaluated using a B-mode ultrasound system (Philips EPIQ 7C, Bothell, United States) equipped with a 5–10 MHz linear array transducer. The transducer was positioned perpendicular to the longitudinal axis of the thigh at a location two-thirds of the distance from the anterior superior iliac spine to the lateral condyle of the knee. After applying a generous amount of coupling gel, quadriceps muscle thickness was measured under both minimal (QT-min) and maximal transducer pressures (QT-max).

For each participant, three consecutive ultrasound images were acquired under each pressure condition. As shown in Supplementary Figure S1, muscle layer thickness includes the rectus femoris and vastus intermedius, depicted in red. All measurements were performed using the ultrasound system’s built-in calipers and recorded in centimeters (cm). For each condition (QT-min and QT-max), the three measurements were averaged for analysis. The measurer was not blinded to patients’ demographic information (e.g., name, age) but was unaware of their clinical status. The intrarater reliability for these measurements was excellent, with an intraclass correlation coefficient (ICC) of 0.97 (*p* < 0.001).

### 2.4 Outcome measures

The primary outcome measure was 28-day mortality after admission, with ventilator duration as a secondary outcome of interest.

### 2.5 Covariates

Demographic characteristics (age, sex, and body mass index [BMI]), Glasgow Coma Scale (GCS) score, Acute Physiology and Chronic Health Evaluation II (APACHE II) score, medical history (including cerebral hemorrhage, cerebral infarction, heart failure, coronary artery disease, pulmonary disease, digestive disorders, malignancies, and prior surgeries), laboratory parameters [total protein (TP), prealbumin, albumin, hemoglobin, aspartate aminotransferase (AST), alanine aminotransferase (ALT), total cholesterol (TC), triglycerides (TG), high-density lipoprotein (HDL), low-density lipoprotein (LDL), and uric acid (UA)] were extracted from electronic medical records at the time of enrollment. Clinical data, including the use of mechanical ventilation, number of organ supports, and vasopressor use, were retrieved from electronic medical records during hospitalization. The number of organ supports was quantified based on interventions administered for the gastrointestinal, cerebrovascular, circulatory, renal, or respiratory systems.

### 2.6 Statistical analysis

The clinical and demographic variables of different groups were compared using the Kruskal–Wallis test for non-parametric variables and the chi-square test for categorical variables. Spearman’s rank correlation was used to assess the correlation be-tween quadriceps thickness and duration of mechanical ventilation.

Cox proportional hazards regression was employed to assess the relationship between quadriceps thickness and time to death, with days from study entry used as the time scale. The results are reported as hazard ratios (HRs) with 95% confidence intervals (CIs). Three predefined multivariable models were constructed. Model 1 was adjusted for age, sex, BMI, and GCS score. In Model 2, we additionally adjusted for variables that differed significantly between groups in univariate analyses, including APACHE II score, duration of mechanical ventilation, number of organ supports, vasopressor use, and serum levels of TP, prealbumin, albumin, and hemoglobin. To address potential overadjustment bias, Model 3 was constructed by removing three variables from Model 2 (duration of mechanical ventilation, number of organ supports, and vasopressor use), as these may reflect post-admission evolution of disease severity. The proportional hazards assumption was tested using Schoenfeld residuals; for Models 1 and 3, all covariates yielded *p* > 0.05. We categorized quadriceps thickness into quartiles (Q1–Q4) and generated Kaplan–Meier curves to compare survival across quartiles.

To evaluate potential effect modification, we included interaction terms for quadriceps mass with sex, age, BMI, mechanical ventilation, number of organ supports, and vasopressor use (i.e., quadriceps mass × sex, quadriceps mass × age, quadriceps mass × BMI, quadriceps mass × mechanical ventilation, quadriceps mass × number of organ supports, and quadriceps mass × vasopressor use) in the Cox regression model adjusted for the same covariates as Model 3. All corresponding main effects were retained.

A 2-tailed *p* < 0.05 was considered significant. Interaction was tested using the Benjamini–Hochberg procedure to control the false discovery rate (FDR), with *q* < 0.05 considered statistically significant. All statistical analyses were performed using R software (version 4.3.3).

## 3 Results

### 3.1 Characteristics of participants

Of the 1,025 patients initially assessed, 422 were excluded before cohort inclusion due to loss to follow-up (*n* = 405), lactation and pre-existing psychiatric disorders (*n* = 5), or incomplete clinical data (*n* = 12). Thus, a total of 603 patients were enrolled in the final analysis cohort and were followed for 28 days (Supplementary Figure S2).

The baseline characteristics of the 603 participants are shown in Table 1. Overall, the mean age was 71.01 (± 14.48) years with 60.10% males. The 28-day mortality rate was 21.06% (127/603). Patients who died were older, had lower BMI, GCS score, and lower levels of total protein, prealbumin, albumin, and hemoglobin, but higher APACHE II score, longer mechanical ventilation time, more organ support, and higher proportion of unstable blood circulation, coronary heart disease and cerebral hemorrhage. In addition, there were significant differences in MUAC, QC, quadriceps thickness measured under minimal transducer pressure (QT-min) and quadriceps thickness measured under maximal transducer pressure (QT-max) between the two study groups in the univariate analysis.

**Table 1:** Baseline characteristics of the participants.

	All (*N* = 603)	Alive (*N* = 476)	Death (*N* = 127)	*P*
Age, year	71.12 (± 14.25)	69.36 (± 14.37)	77.72 (± 11.66)	<0.0010
Sex, male	363 (60.20%)	293 (61.55%)	70 (55.12%)	0.2348
BMI (kg/m^2^)	23.57(±4.31)	23.99 (± 4.24)	21.99 (± 4.245)	<0.0010
GCS score	13.09 (± 3.18)	13.75 (± 2.59)	10.61 (± 3.906)	<0.0010
APACHE II score	19.64 (± 4.40)	18.40 (± 3.73)	24.28 (± 3.521)	<0.0010
Mechanical ventilation	150 (24.83%)	84 (17.61%)	66 (51.97%)	<0.0010
Non-invasive	113 (18.714%)	71 (14.92%)	42 (33.07%)	
Invasive	41 (6.80%)	15 (3.15%)	26 (20.47%)	
Duration, day	2.23 (± 5.57)	1.714 (± 5.21)	4.165 (± 6.395)	<0.0010
Organ supports, n	0.40 (± 0.64)	0.24 (± 0.47)	1.031 (± 0.7862)	<0.0010
Vasopressor use	89 (14.74%)	40 (8.40%)	49 (38.58%)	<0.0010
TP, g/L	65.57 (± 8.80)	66.29 (± 8.79)	62.85 (± 8.312)	<0.0010
Prealbumin, g/L	0.17 (± 0.13)	0.18 (± 0.12)	0.1349 (± 0.1530)	<0.0010
Albumin, g/L	37.93 (± 6.01)	38.64 (± 5.95)	35.30 (± 5.493)	<0.0010
Hemoglobin	119.20 (± 32.56)	122.40 (± 32.22)	107.4 (± 31.22)	<0.0010
AST, U/L	47.77 (± 134.70)	49.50 (± 149.20)	41.06 (± 52.02)	0.6708
ALT, U/L	35.66 (± 114.40)	37.66 (± 127.30)	27.89 (± 37.76)	0.1037
TC, mmol/L	4.01 (± 1.47)	4.04 (± 1.51)	3.881 (± 1.304)	0.1937
TG, mmol/L	1.60 (± 1.77)	1.64 (± 1.93)	1.434 (± 0.9087)	0.1843
HDL, mmol/L	0.98 (± 0.40)	1.00 (± 0.39)	0.9329 (± 0.4231)	0.1409
LDL, mmol/L	2.50 (± 1.14)	2.52 (± 1.15)	2.410 (± 1.106)	0.2555
UA, mmol/L	409.30 (± 313.90)	402.40(± 330.70)	435.3 (± 240.0)	0.0700
MUAC	25.63 (± 4.02)	26.16 (± 3.85)	23.65 (± 4.02)	<0.0010
QC	43.85 (± 6.52)	44.79 (± 6.17)	40.36 (± 6.644)	<0.0010
QT-min	2.27 (± 0.70)	2.44 (± 0.70)	1.63 (± 0.55)	<0.0010
QT-max	1.17 (± 0.50)	1.24 (± 0.51)	0.86 (± 0.30)	<0.0010
History				
Cerebral hemorrhage	23 (3.81%)	16 (3.36%)	7 (5.51%)	0.3853
Cerebral infarction	101 (16.75%)	71 (14.92%)	30 (23.62%)	0.0270
Heart failure	78 (12.94%)	55 (11.55%)	23 (18.11%)	0.0694
Coronary disease	207 (34.33%)	150 (31.51%)	57 (44.88%)	0.0063
Pulmonary disease	87 (14.43%)	70 (14.71%)	17 (13.39%)	0.8216
Digestive disorders	46 (7.63%)	42 (8.82%)	4 (3.15%)	0.0515
Tumor	74 (12.27%)	54 (11.34%)	20 (15.75%)	0.2301
Operation	28 (4.64%)	21 (4.41%)	7 (5.51%)	0.6174

BMI, body mass index; GCS, Glasgow Coma Scale; APACHE II, Acute Physiology and Chronic Health Evaluation II; TP, total protein; AST, aspartate aminotransferase; ALT, alanine aminotransferase; TC, total cholesterol; TG, triglycerides; HDL, high-density lipoprotein; LDL, low-density lipoprotein; UA, uric acid; MUAC, mid-upper arm circumference; QC, quadriceps circumference; QT-min, quadriceps thickness measured under minimal transducer pressure; QT-max, quadriceps thickness measured under maximal transducer pressure. Data are presented as mean (± SD) for continuous variables and n (%) for categorical variables.

### 3.2 Relationship between muscle mass and mortality

In the Cox regression analyses including all 603 patients, 127(21.06%) died within 28 days. As shown in Table 2, MUAC, quadriceps circumference, and quadriceps thickness were protective factors for death in the univariate and Model 1 Cox regression analyses (*p* < 0.05). In the Model 2 Cox regression analysis, quadriceps circumference (HR 0.95; 95% CI 0.91–1.00, *p* = 0.0381), QT-min (HR 0.59; 95% CI 0.39–0.91, *p* = 0.0172), QT-max (HR 0.47; 95% CI 0.22–0.98, *p* = 0.0452) were protective factors for death. No significant association was observed between MUAC and death (*p* = 0.5852). In Model 3, the protective effects of a 1-cm increase in quadriceps circumference (HR 0.95; 95% CI 0.91–1.00, *p* = 0.0119), QT-min (HR 0.63; 95% CI 0.42–0.92, *p* = 0.0183), and QT-max (HR 0.42; 95% CI 0.20–0.85, *p* = 0.0169) persisted, while MUAC remained non-significant (HR 0.88; 95% CI 0.91–1.01, *p* = 0.1235).

**Table 2:** Cox regression analysis of muscle mass for 28-day mortality.

	Unadjusted	*P*	Modle1	*P*	Modle2	*P*	Modle3	*P*
	HR (95%IC)		HR (95%IC)		HR (95%IC)		HR (95%IC)	
MUAC	0.87(0.84–0.91)	0.000	0.89(0.84–0.95)	0.0007	0.98(0.91–1.05)	0.5852	0.88(0.91–1.01)	0.1235
QC	0.92(0.90–0.94)	0.000	0.93(0.89–0.97)	0.0003	0.95(0.91–1.00)	0.0381	0.95(0.91–1.00)	0.0119
QT-min	0.34(0.26–0.44)	0.000	0.42(0.29–0.60)	0.000	0.59(0.39–0.91)	0.0172	0.63(0.42–0.92)	0.0183
QT-max	0.18(0.11–0.29)	0.000	0.21(0.11–0.42)	0.000	0.47(0.22–0.98)	0.0452	0.42(0.20–0.85)	0.0169

MUAC, mid-upper arm circumference; QC, quadriceps circumference; QT-min, quadriceps thickness measured under minimal transducer pressure; QT-max, quadriceps thickness measured under maximal transducer pressure; HR, hazard ratio; CI, confidence interval; P, p-value. Model 1 was adjusted for age, sex, BMI, and GCS score; Model 2 included all variables in Model 1 plus APACHE II score, duration of mechanical ventilation, number of organ supports, vasopressor use, and serum levels of TP, prealbumin, albumin, and hemoglobin; Model 3 included all variables in Model 1 plus APACHE II score, serum levels of TP, prealbumin, albumin, and hemoglobin.

**Figure 1:**
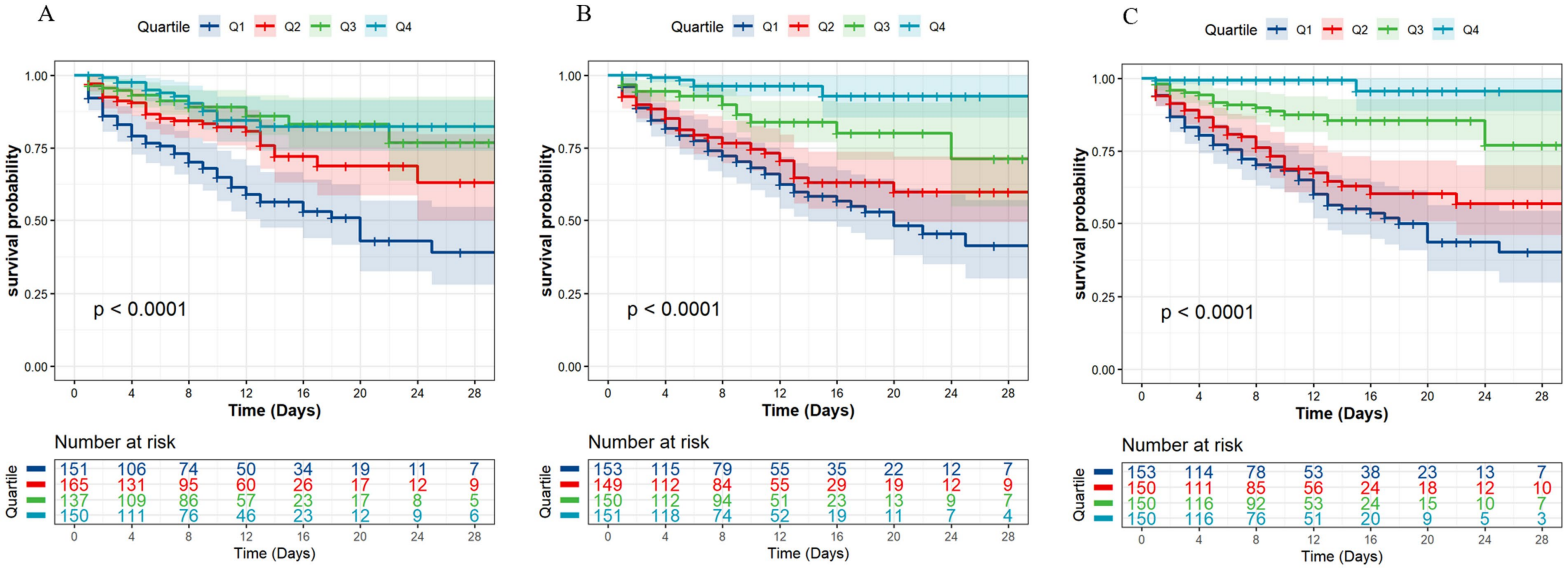
Kaplan–Meier survival plot for quartiles of quadriceps circumference and thickness. **(A)** Quadriceps circumference. **(B)** Quadriceps thickness measured under maximal transducer pressure. **(C)** Quadriceps thickness measured under minimal transducer pressure.

For illustrative purposes, we plotted Kaplan–Meier survival curves based on quartiles of quadriceps circumference and thickness (Figure 1). Patients with higher quadriceps circumference and thickness had considerably longer overall survival than those with lower values.

### 3.3 Stratification analyses

**Figure 2:**
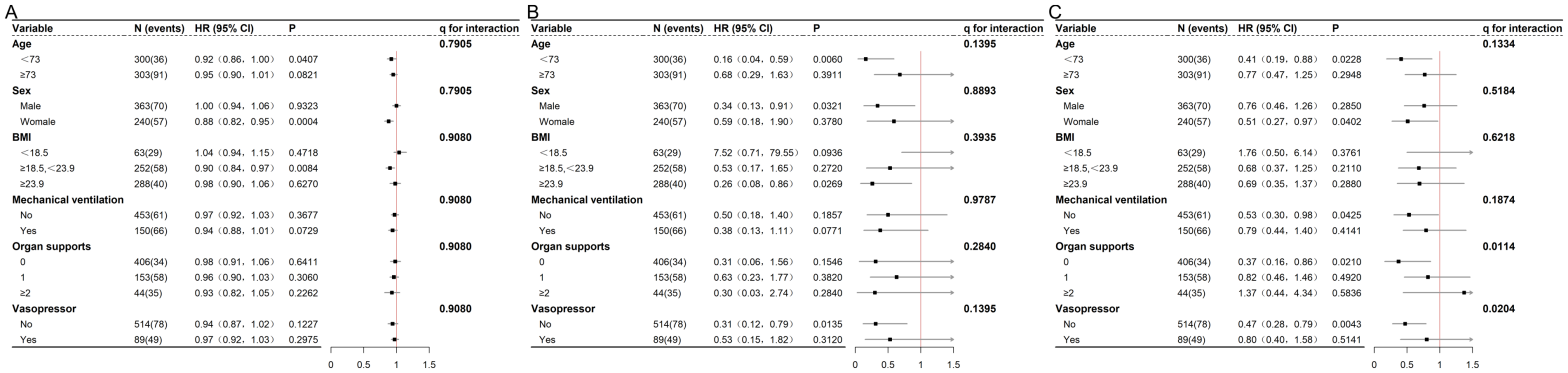
Subgroup analysis of the association between quadriceps mass and 28-day mortality. Data are presented as number of patients per subgroup (N) with number of deaths (events). Hazard ratios (HRs) and 95% confidence intervals (CIs) were calculated using Cox proportional hazards model 3. *q*: FDR-adjusted *P* for interaction. **(A)** Quadriceps circumference. **(B)** Quadriceps thickness measured under maximal transducer pressure. **(C)** Quadriceps thickness measured under minimal transducer pressure.

After FDR correction, significant interactions were found for QT-min with organ supports (*q* = 0.0114) and vasopressor use (*q* = 0.0204). The protective effect of QT-min was more pronounced in patients without organ supports (HR 0.37; 95% CI 0.16–0.86) and in those without vasopressor use (HR 0.47; 95% CI 0.28–0.79) (Figure 2).

### 3.4 Associations of quadriceps mass with mechanical ventilation duration

**Figure 3:**
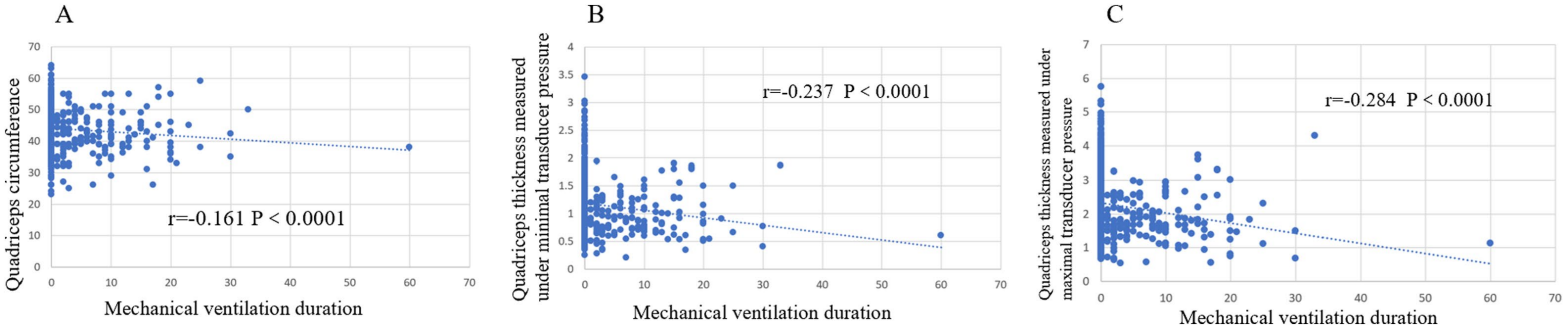
Correlation between quadriceps mass with and mechanical ventilation duration. **(A)** Quadriceps circumference. **(B)** Quadriceps thickness measured under maximal transducer pressure. **(C)** Quadriceps thickness measured under minimal transducer pressure.

There was a statistically significant correlation between QC, QT-max, QT-min and mechanical ventilation duration (*p* < 0.0001). The Spearman correlation coefficients were −0.161, −0.237, and −0.284, respectively (Figure 3).

## 4 Discussion

Our findings indicate that a low quadriceps mass, whether evaluated by circumferential measurement or ultrasound-based thickness, is independently associated with increased mortality in critically ill patients. This relationship remained robust across most patient subgroups, except in patients with vasopressor use or those requiring at least one organ support, where QT-min showed no significant effect. Moreover, among patients who were mechanically ventilated, a reduced quadriceps mass was associated with prolonged mechanical ventilation.

Consistent with our findings, several studies have reported a strong association between low muscle mass and mortality using CT assessment. For example, a study of 240 critically ill patients found that lower skeletal muscle volume at L3 on admission CT scans independently predicted higher mortality, regardless of APACHE II scores or gender, while BMI showed no correlation with outcomes (8). Similarly, Ji et al. reported intra-abdominal sarcopenic obesity, assessed by CT via the skeletal muscle index and visceral adipose tissue area, was associated with increased 30-day mortality (17). Further supporting this, systematic reviews and meta-analysis revealed a significant association between low skeletal muscle mass (LSMM) and increased mortality risk in critically ill patients (9, 18). Additionally, higher skeletal muscle density measured by CT has also been associated with longer postoperative survival (19–21).

Recent ultrasound-based investigations have reproduced these findings across diverse critical care populations. For instance, in a recent study by Lim et al., greater quadriceps muscle thickness measured by ultrasound at ICU admission was positively correlated with physical function outcomes at hospital discharge (22). Hadda et al. reported that loss of muscle thickness assessed by ultrasound predicted poor prognosis in sepsis patients (23). Similarly, Toledo et al. identified that significant muscle wasting in mechanically ventilated critically ill patients, with greater loss of quadriceps muscle layer thickness, was associated with worse clinical outcomes (24). The prognostic relevance of muscle mass has also been emphasized in idiopathic pulmonary fibrosis (25). Moreover, ultrasound-derived measures of muscle thickness and related indices have been linked to mortality in various clinical contexts, including decompensated liver cirrhosis (26), hemodialysis (27, 28), cancer (29), and heart failure (30). These findings, along with our results, reinforce that the role of muscle assessment as a crucial physiological biomarker and a valuable prognostic tool across a spectrum of patient populations. This notion is further supported by recent studies demonstrating that the low skeletal muscle index significantly increases all-cause mortality risk in elderly (31) and adult populations (32).

In previous ultrasound studies assessing muscle mass in critically ill patients, methodological approaches have varied considerably. Some researchers have applied full probe compression to mitigate edema effects, though this may alter muscle dimensions (33). Others use minimal compression with generous gel to avoid tissue distortion (34). Such methodological heterogeneity—particularly the use of full versus minimal transducer pressure—hinders cross-study comparability and underscores the need for standardized reporting of pressure protocols (15). A key methodological innovation of this study is the simultaneous application of two measurement methods (QT-min and QT-max) to assess muscle thickness in the same patient cohort. While QT-min reflects uncompressed anatomical muscle thickness, QT-max may offer a parameter less influenced by edema, fibrosis, or fatty infiltration. Both methods independently predicted mortality in fully adjusted models, with QT-max showing a stronger numerical effect (0.42 vs. 0.63 HR per 1-cm increase). To our knowledge, this is the first study to systematically compare these two ultrasound techniques in critically ill patients.

Furthermore, the protective effect of higher QT-min was more pronounced in patients without organ supports and in those not requiring vasopressors. These findings suggest that the prognostic value of muscle thickness may be modified by the severity of organ dysfunction and hemodynamic instability. In patients with multi-organ failure or vasopressor dependence, competing mortality risks from acute circulatory collapse may overshadow the contribution of muscle mass. Moreover, muscle ultrasound measurements are susceptible to confounding by edema and tissue quality (15), and fluid resuscitation in shock states can artificially increase muscle thickness, thereby attenuating its prognostic accuracy (35). In our study, an interaction was observed only for QT-min. We hypothesize that QT-max may better reflect true muscle thickness by reducing the influence of edema and other tissue alterations, potentially accounting for the absence of subgroup differences with this measurement. We acknowledge that the sample sizes for patients receiving organ support (1 organ support: *n* = 153; ≥2 organ supports: *n* = 44) and those on vasopressors (*n* = 89) were relatively small, which may have limited statistical power to detect true associations and increased the risk of type II error. Therefore, the observed effect modification should be interpreted cautiously and larger multicenter studies are warranted to confirm these findings.

The mechanism underlying the association between reduced muscle thickness and mortality in critically ill patients may involve metabolic disorders, systemic inflammation, and disuse. The inflammatory response in critical illness accelerates muscle wasting (36). Specifically, inflammatory mediators such as Interleukin-6 and tumor necrosis factor-alpha increase protein breakdown and suppress protein synthesis in muscle tissue (37), ultimately leading to functional decline and increased mortality risk. Additionally, microcirculatory dysfunction with impaired oxygen delivery (38), mitochondrial bioenergetic failure leading to reduced ATP production (39), and disruption of membrane ion channels collectively contribute to muscle dysfunction in critical illness (40).

In our study, the association between mid-upper arm circumference (MUAC) and mortality was attenuated after adjusting for risk factors, whereas QC and quadriceps ultrasound-derived thickness remained independently associated with mortality. This differential performance may reflect the preferential atrophy of lower limb muscles in bedridden ICU patients (41), making quadriceps measures more sensitive to acute wasting. These findings support current recommendations favoring lower limb assessment for muscle monitoring in critical care (42).

### 4.1 Limitations

There are some limitations in this study. First, the observed association between low quadriceps thickness and mortality may be subject to reverse causation and residual confounding. Low muscle mass could reflect either pre-existing frailty or acute wasting, and despite extensive adjustment for APACHE II score and other severity markers, unmeasured confounding remains possible. Patients with greater physiological instability may have both lower muscle mass and higher mortality due to factors not fully captured by our covariates. Second, muscle measurements were obtained only at admission. Serial measurements would have allowed us to evaluate the prognostic value of muscle change over time. Third, we did not stratify patients by specific diagnoses or document causes of death, precluding disease-specific inferences. Fourth, as a single-center study, our findings require validation through larger, multicenter investigations.

### 4.2 Future directions

Larger studies with more diverse populations are needed to validate our findings and determine the generalizability of QT-min and QT-max as prognostic tools across different ICU settings and disease conditions. Whether early nutritional support or resistance exercise can mitigate muscle wasting and thereby improve outcomes in high-risk patients warrants further investigation through interventional studies.

## 5 Conclusion

In conclusion, baseline quadriceps muscle mass at admission is independently associated with mortality in critically ill patients, with a protective effect that remained consistent across most subgroups. However, the prognostic value of QT-min was significant only in hemodynamically stable patients and those without organ support. Given the small sample sizes in specific subgroups, particularly among patients receiving vasopressors (*n* = 89) or multiple organ supports (*n* = 44), these interaction findings should be interpreted with caution due to limited statistical power. As MUAC lost prognostic significance in multivariable models, our study supports the use of quadriceps ultrasound and manual measurement as practical tools for the early identification of high-risk individuals.

## Data Availability

The raw data supporting the conclusions of this article will be made available by the authors, without undue reservation.

## Supplementary material

Supplementary Figure S1 Illustrations of muscle thickness measured by ultrasonography. The scan shows the rectus femoris and vastus intermedius (outlined in red) in a 42-year-old ICU female with a body mass index of 20.70 kg/m². **(A)** Measurement obtained with maximal transducer pressure. **(B)** Measurement obtained with minimal transducer pressure.

Supplementary Figure S2 Patient enrollment flowchart. Of the 1,025 patients initially assessed, 422 were excluded prior to cohort inclusion due to loss to follow-up, lactation and pre-existing psychiatric disorders, or incomplete data. Consequently, 603 patients were included in the final analysis cohort, all of whom completed the 28-day follow-up.

## References

[1] Ferrando AA Paddon-Jones D Wolfe RR. Bed rest and myopathies. Curr Opin Clin Nutr Metab Care. (2006) 9:410–5. doi: 10.1097/01.mco.0000232901.59168.e9, 16778570

[2] Puthucheary ZA Rawal J McPhail M Connolly B Ratnayake G Chan P . Acute skeletal muscle wasting in critical illness. JAMA. (2013) 310:1591. doi: 10.1001/jama.2013.278481, 24108501

[3] Fazzini B Märkl T Costas C Blobner M Schaller SJ Prowle J . The rate and assessment of muscle wasting during critical illness: a systematic review and meta-analysis. Crit Care. (2023) 27:2. doi: 10.1186/s13054-022-04253-0, 36597123 PMC9808763

[4] Preiser J-C Arabi YM Berger MM Casaer M McClave S Montejo-González JC . A guide to enteral nutrition in intensive care units: 10 expert tips for the daily practice. Crit Care. (2021) 25:424. doi: 10.1186/s13054-021-03847-4, 34906215 PMC8669237

[5] Looijaard WGPM Dekker IM Beishuizen A Girbes ARJ Oudemans-van Straaten HM Weijs PJM. Early high protein intake and mortality in critically ill ICU patients with low skeletal muscle area and -density. Clin Nutr. (2020) 39:2192–201. doi: 10.1016/j.clnu.2019.09.007, 31669003

[6] L B A S N R J V. The impact of guideline recommended protein intake on mortality and length of intensive care unit and hospital stay in critically ill adults: a systematic review. Clin Nutr ESPEN. (2024) 61:356–8. doi: 10.1016/j.clnesp.2024.04.003 38777455

[7] Koga Y Fujita M Yagi T Todani M Nakahara T Kawamura Y . Early enteral nutrition is associated with reduced in-hospital mortality from sepsis in patients with sarcopenia. J Crit Care. (2018) 47:153–8. doi: 10.1016/j.jcrc.2018.06.026 29990793

[8] Weijs PJM Looijaard WGPM Dekker IM Stapel SN Girbes AR van Oudemans- Straaten HM . Low skeletal muscle area is a risk factor for mortality in mechanically ventilated critically ill patients. Crit Care. (2014) 18:R12. doi: 10.1186/cc13189, 24410863 PMC4028783

[9] Yang H Wan X-X Ma H Li Z Weng L Xia Y . Prevalence and mortality risk of low skeletal muscle mass in critically ill patients: an updated systematic review and meta-analysis. Front Nutr. (2023) 10:1117558. doi: 10.3389/fnut.2023.1117558, 37252244 PMC10213681

[10] Connolly B MacBean V Crowley C Lunt A Moxham J Rafferty GF . Ultrasound for the assessment of peripheral skeletal muscle architecture in critical illness: a systematic review. Crit Care Med. (2015) 43:897–905. doi: 10.1097/CCM.0000000000000821 25559437

[11] Nakanishi N Tsutsumi R Okayama Y Takashima T Ueno Y Itagaki T . Monitoring of muscle mass in critically ill patients: comparison of ultrasound and two bioelectrical impedance analysis devices. J Intensive Care. (2019) 7:61. doi: 10.1186/s40560-019-0416-y, 31890223 PMC6916000

[12] Casey P Alasmar M McLaughlin J Ang Y McPhee J Heire P . The current use of ultrasound to measure skeletal muscle and its ability to predict clinical outcomes: A systematic review. J Cachexia Sarcopenia Muscle. (2022) 13:2298–309. doi: 10.1002/jcsm.13041, 35851996 PMC9530572

[13] Sabatino A Regolisti G Bozzoli L Fani F Antoniotti R Maggiore U . Reliability of bedside ultrasound for measurement of quadriceps muscle thickness in critically ill patients with acute kidney injury. Clin Nutr. (2017) 36:1710–5. doi: 10.1016/j.clnu.2016.09.029, 27743614

[14] Rodriguez C Mota JD Palmer TB Heymsfield SB Tinsley GM. Skeletal muscle estimation: a review of techniques and their applications. Clin Physiol Funct Imaging. (2024) 44:261–84. doi: 10.1111/cpf.12874, 38426639

[15] Mourtzakis M Parry S Connolly B Puthucheary Z. Skeletal muscle ultrasound in critical care: A tool in need of translation. Ann Am Thorac Soc. (2017) 14:1495–503. doi: 10.1513/AnnalsATS.201612-967PS, 28820608 PMC5718569

[16] Heckmatt JZ Leeman S Dubowitz V. Ultrasound imaging in the diagnosis of muscle disease. J Pediatr. (1982) 101:656–60. doi: 10.1016/s0022-3476(82)80286-2, 7131136

[17] Ji Y Cheng B Xu Z Ye H Lu W Luo X . Impact of sarcopenic obesity on 30-day mortality in critically ill patients with intra-abdominal sepsis. J Crit Care. (2018) 46:50–4. doi: 10.1016/j.jcrc.2018.03.019, 29677586

[18] Su H Ruan J Chen T Lin E Shi L. CT-assessed sarcopenia is a predictive factor for both long-term and short-term outcomes in gastrointestinal oncology patients: a systematic review and meta-analysis. Cancer Imaging. (2019) 19:82. doi: 10.1186/s40644-019-0270-0, 31796090 PMC6892174

[19] Nie T Wu F Heng Y Cai W Liu Z Qin L . Influence of skeletal muscle and intermuscular fat on postoperative complications and long-term survival in rectal cancer patients. J Cachexia Sarcopenia Muscle. (2024) 15:702–17. doi: 10.1002/jcsm.13424, 38293722 PMC10995272

[20] Raoul P Cintoni M Coppola A Alfieri S Tortora G Gasbarrini A . Preoperative low skeletal muscle mass index assessed using L3-CT as a prognostic marker of clinical outcomes in pancreatic cancer patients undergoing surgery: a systematic review and meta-analysis. Int J Surg. (2024) 110:6126–34. doi: 10.1097/JS9.0000000000000989, 38836800 PMC11486987

[21] Wang S Wang M Jiang L Zhao X. Low skeletal muscle quality extracted from CT is associated with poor outcomes in severe acute pancreatitis patients. Eur J Radiol. (2024) 170:111215. doi: 10.1016/j.ejrad.2023.111215, 38091663

[22] Lim SY Park JS Cho Y-J Lee JH Lee C-T Lee YJ. Association of baseline muscle mass with functional outcomes in intensive care unit survivors: A single-center retrospective cohort study in Korea. Medicine (Baltimore). (2024) 103:e39156. doi: 10.1097/MD.0000000000039156, 39121260 PMC11315508

[23] Hadda V Kumar R Khilnani GC Kalaivani M Madan K Tiwari P . Trends of loss of peripheral muscle thickness on ultrasonography and its relationship with outcomes among patients with sepsis. J Intensive Care. (2018) 6:81. doi: 10.1186/s40560-018-0350-4, 30564367 PMC6292013

[24] Toledo DO Freitas BJD Dib R Pfeilsticker FJDA Santos DMD Gomes BC . Peripheral muscular ultrasound as outcome assessment tool in critically ill patients on mechanical ventilation: an observational cohort study. Clin Nutr ESPEN. (2021) 43:408–14. doi: 10.1016/j.clnesp.2021.03.015, 34024548

[25] Fernández-Jiménez R Cabrera Cesar E Sánchez García A Espíldora Hernández F Vegas-Aguilar IM Amaya-Campos MDM . Rectus Femoris cross-sectional area and phase angle asPredictors of 12-month mortality in idiopathic pulmonary fibrosis patients. Nutrients. (2023) 15:4473. doi: 10.3390/nu15204473, 37892547 PMC10609753

[26] Hari A Berzigotti A Štabuc B Caglevič N. Muscle psoas indices measured by ultrasound in cirrhosis — preliminary evaluation of sarcopenia assessment and prediction of liver decompensation and mortality. Dig Liver Dis. (2019) 51:1502–7. doi: 10.1016/j.dld.2019.08.017, 31547952

[27] Sabatino A Kooman JP Di Motta T Cantarelli C Gregorini M Bianchi S . Quadriceps muscle thickness assessed by ultrasound is independently associated with mortality in hemodialysis patients. Eur J Clin Nutr. (2022) 76:1719–26. doi: 10.1038/s41430-022-01166-7, 35641665

[28] Sabatino A Kooman J Avesani CM Gregorini M Bianchi S Regolisti G . Sarcopenia diagnosed by ultrasound-assessed quadriceps muscle thickness and handgrip strength predicts mortality in patients on hemodialysis. J Nephrol. (2024) 37:993–1003. doi: 10.1007/s40620-023-01867-7, 38263531

[29] García-García C Vegas-Aguilar IM Rioja-Vázquez R Cornejo-Pareja I Tinahones FJ García-Almeida JM. Rectus Femoris muscle and phase angle as prognostic factor for 12-month mortality in a longitudinal cohort of patients with Cancer (AnyVida trial). Nutrients. (2023) 15:522. doi: 10.3390/nu15030522, 36771229 PMC9919732

[30] Saito H Fujimoto Y Matsue Y Yoshioka K Maekawa E Kamiya K . Ultrasound-measured quadriceps muscle thickness and mortality in older patients with heart failure. Can J Cardiol. (2024) 40:2555–64. doi: 10.1016/j.cjca.2024.09.007 39270750

[31] Li C-I Liu C-S Lin C-H Yang S-Y Li T-C Lin C-C. Independent and joint associations of skeletal muscle mass and physical performance with all-cause mortality among older adults: a 12-year prospective cohort study. BMC Geriatr. (2022) 22:597. doi: 10.1186/s12877-022-03292-0, 35850584 PMC9295364

[32] Cheng Y Li T Huang G Hou D Li S Liang Y . Low appendicular skeletal muscle mass is associated with the risk of mortality among adults in the United States. Sci Rep. (2025) 15:9908. doi: 10.1038/s41598-025-94357-8, 40121321 PMC11929809

[33] Paris MT Mourtzakis M Day A Leung R Watharkar S Kozar R . Validation of bedside ultrasound of muscle layer thickness of the quadriceps in the critically ill patient (VALIDUM study): a prospective multicenter study. J Parenter Enter Nutr. (2017) 41:171–80. doi: 10.1177/0148607116637852, 26962061

[34] Puthucheary ZA Phadke R Rawal J McPhail MJW Sidhu PS Rowlerson A . Qualitative ultrasound in acute critical illness muscle wasting. Crit Care Med. (2015) 43:1603–11. doi: 10.1097/CCM.0000000000001016, 25882765

[35] Skoczynski R Hansen J Adhikary SD Lehman E Bonavia AS. Point-of-care ultrasound as a prognostic tool in critically ill patients: insights beyond core muscle mass. Ther Adv Pulm Crit Care Med. (2025) 20:29768675251397475. doi: 10.1177/29768675251397475, 41321899 PMC12660653

[36] Vesali RF Cibicek N Jakobsson T Klaude M Wernerman J Rooyackers O. Protein metabolism in leg muscle following an endotoxin injection in healthy volunteers. Clin Sci (Lond). (2009) 118:421–7. doi: 10.1042/CS20090332, 19751216

[37] van Hall G. Cytokines: muscle protein and amino acid metabolism. Curr Opin Clin Nutr Metab Care. (2012) 15:85–91. doi: 10.1097/MCO.0b013e32834e6ea2, 22123617

[38] Hepple RT. Skeletal muscle: microcirculatory adaptation to metabolic demand. Med Sci Sports Exerc. (2000) 32:117–23. doi: 10.1097/00005768-200001000-00018, 10647538

[39] Di Marco G Gherardi G De Mario A Piazza I Baraldo M Mattarei A . The mitochondrial ATP-dependent potassium channel (mitoKATP) controls skeletal muscle structure and function. Cell Death Dis. (2024) 15:58. doi: 10.1038/s41419-024-06426-x, 38233399 PMC10794173

[40] Liu M Chen Y-T Wang G-L Wu X-M. Risk factors for intensive-care-unit-acquired weakness. World J Clin Cases. (2024) 12:4853–5. doi: 10.12998/wjcc.v12.i21.4853, 39070851 PMC11235492

[41] Turton P Hay R Taylor J McPhee J Welters I. Human limb skeletal muscle wasting and architectural remodeling during five to ten days intubation and ventilation in critical care – an observational study using ultrasound. BMC Anesthesiol. (2016) 16:119. doi: 10.1186/s12871-016-0269-z, 27894277 PMC5127036

[42] Venco R Artale A Formenti P Deana C Mistraletti G Umbrello M. Methodologies and clinical applications of lower limb muscle ultrasound in critically ill patients: A systematic review and meta-analysis. Ann Intensive Care. (2024) 14:163. doi: 10.1186/s13613-024-01395-y, 39443352 PMC11499498

