# Supplementary figures and images for: Ultrasound- and circumference-based quadriceps mass is an independent predictor of 28-day mortality in critically ill patients
Source: Front Nutr. 2026 Apr 7;13:1751365. doi: 10.3389/fnut.2026.1751365 (PMC13095706; doi:10.3389/fnut.2026.1751365)

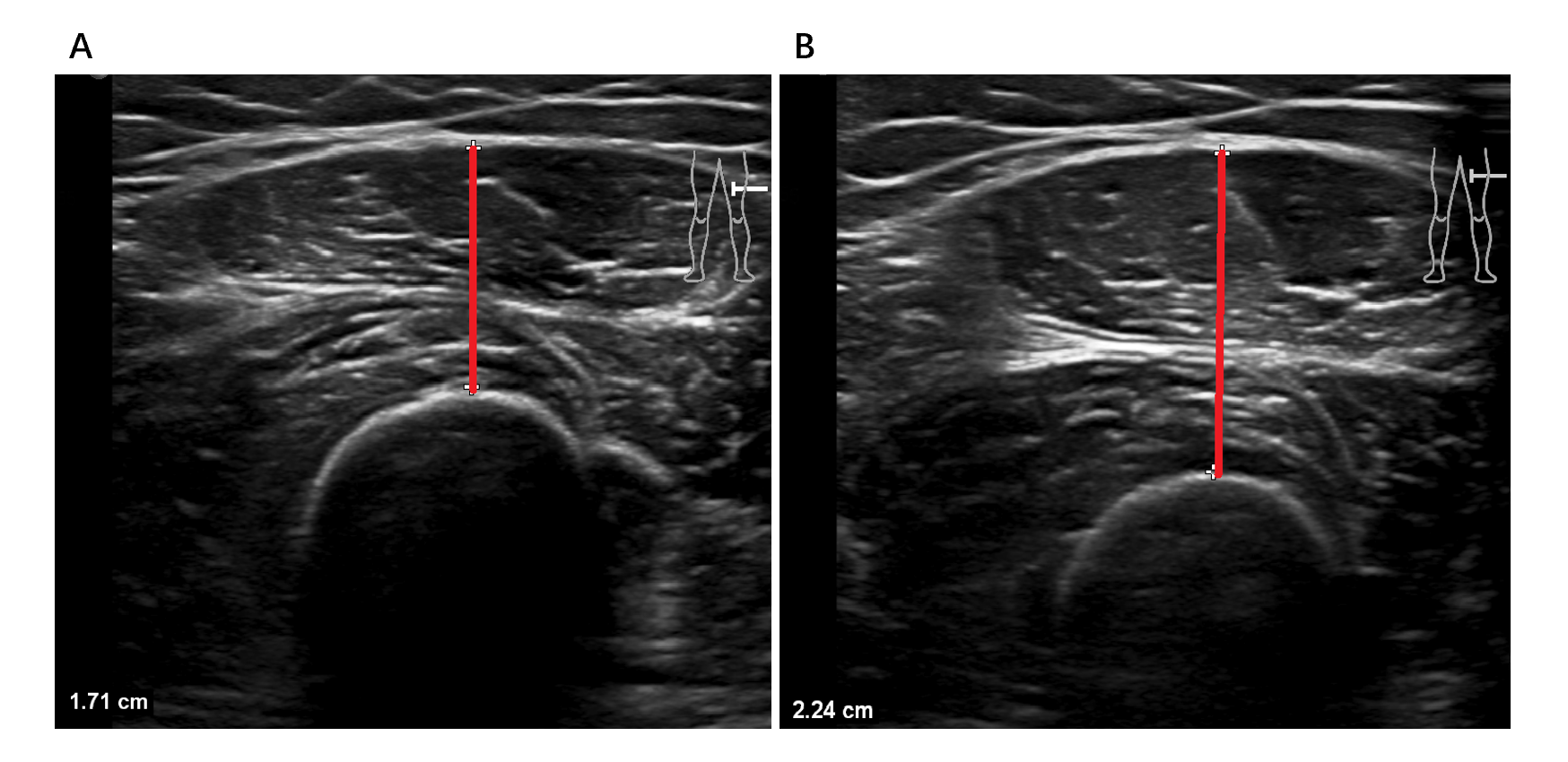

Supplement: Supplementary Figure S1 — Illustrations of muscle thickness measured by ultrasonography. The scan shows the rectus femoris and vastus intermedius (outlined in red) in a 42-year-old ICU female with a body mass index of 20.70 kg/m². (A) Measurement obtained with maximal transducer pressure. (B) Measurement obtained with minimal transducer pressure. [file Image_1.TIFF]

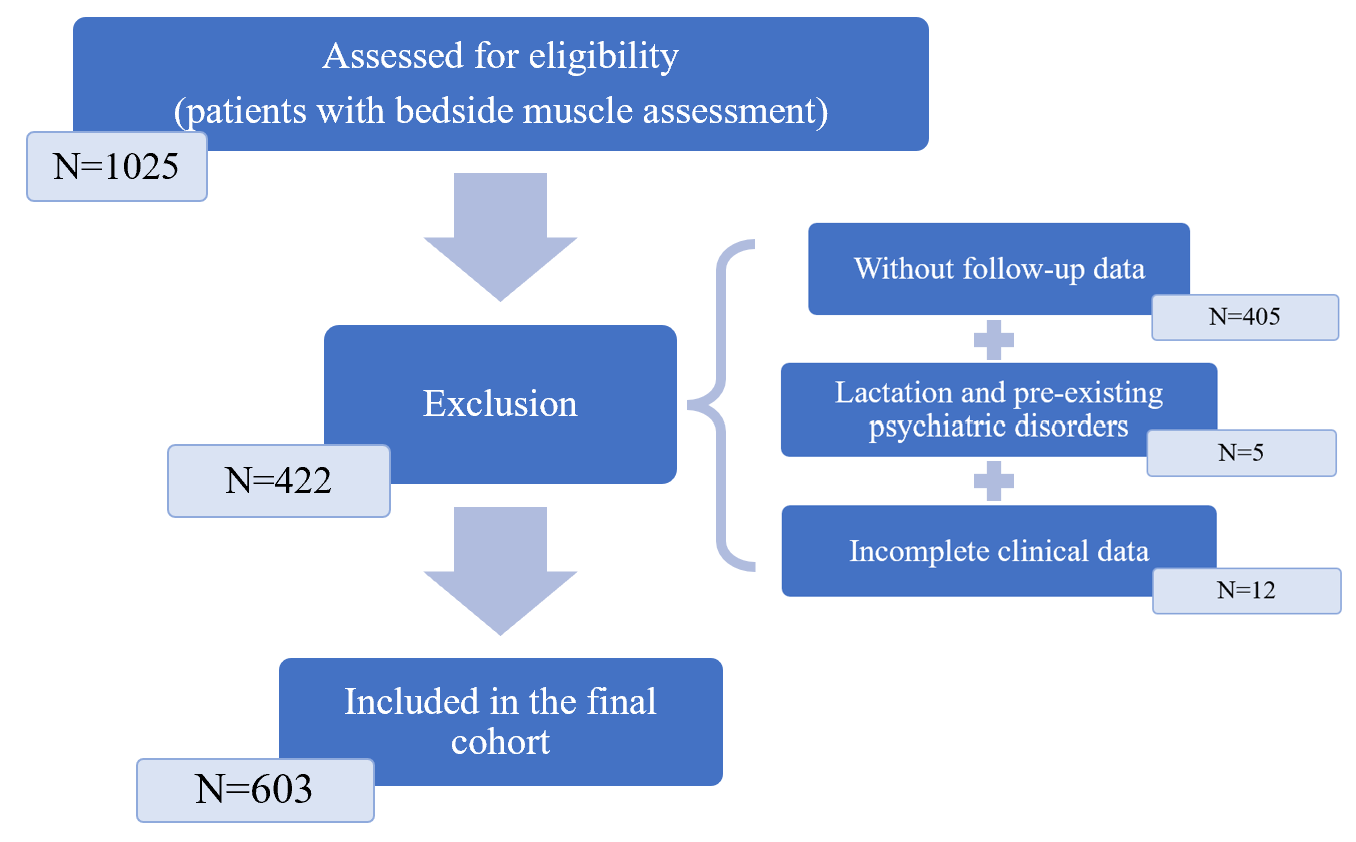

Supplement: Supplementary Figure S2 — Patient enrollment flowchart. Of the 1,025 patients initially assessed, 422 were excluded prior to cohort inclusion due to loss to follow-up, lactation and pre-existing psychiatric disorders, or incomplete data. Consequently, 603 patients were included in the final analysis cohort, all of whom completed the 28-day follow-up. [file Image_2.TIFF]
